# Is the narrative the message? The relationship between suicide-related narratives in media reports and subsequent suicides

**DOI:** 10.1177/00048674221117072

**Published:** 2022-08-23

**Authors:** Lance L Hawley, Thomas Niederkrotenthaler, Rabia Zaheer, Ayal Schaffer, Donald A Redelmeier, Anthony J Levitt, Jitender Sareen, Jane Pirkis, Mark Sinyor

**Affiliations:** 1 Frederick W. Thompson Anxiety Disorders Centre, Sunnybrook Health Sciences Centre, Toronto, ON, Canada; 2 Department of Psychiatry, Sunnybrook Health Sciences Centre, University of Toronto, Toronto, ON, Canada; 3 Unit Suicide Research and Mental Health Promotion, Department of Social and Preventive Medicine, Center for Public Health, Medical University of Vienna, Vienna, Austria; 4 Department of Medicine, University of Toronto, Toronto, ON, Canada; 5 Evaluative Clinical Sciences, Sunnybrook Research Institute, Toronto, ON, Canada; 6 Division of General Internal Medicine, Sunnybrook Health Sciences Centre, Toronto, ON, Canada; 7 Institute for Clinical Evaluative Sciences, Toronto, ON, Canada; 8 Departments of Psychiatry, Psychology and Community Health Sciences, University of Manitoba, Winnipeg, MB, Canada; 9 Centre for Mental Health, Melbourne School of Population and Global Health, The University of Melbourne, Melbourne, VIC, Australia

**Keywords:** Suicide, media, media narrative, Werther effect, Papageno effect

## Abstract

**Objectives:**

When journalists report on the details of a suicide, the way that they contextualize the meaning of the event (i.e. the ‘narrative’) can have significant consequences for readers. The ‘Werther’ and ‘Papageno’ narrative effects refer to increases and decreases in suicides across populations following media reports on suicidal acts or mastery of crises, respectively. The goal of this study was to investigate the impact of these different narrative constructs on subsequent suicides.

**Methods:**

This study examined the change in suicide counts over time in Toronto, Canada. It used latent difference score analysis, examining suicide-related print media reports in the Toronto media market (2011–2014). Articles (*N* = 6367) were coded as having a potentially harmful narrative if they described suicide in a celebrity or described a suicide death in a non-celebrity and included the suicide method. Articles were coded as having potentially protective narratives if they included at least one element of protective content (e.g. alternatives to suicide) without including any information about suicidal behaviour (i.e. suicide attempts or death).

**Results:**

Latent difference score longitudinal multigroup analyses identified a dose–response relationship in which the trajectory of suicides following harmful ‘Werther’ narrative reports increased over time, while protective ‘Papageno’ narrative reports declined. The latent difference score model demonstrated significant goodness of fit and parameter estimates, with each group demonstrating different trajectories of change in reported suicides over time: (χ^2^[6], *N* = 6367) = 13.16; χ^2^/df = 2.19; Akaike information criterion = 97.16, comparative fit index = 0.96, root mean square error of approximation = 0.03.

**Conclusion:**

Our findings support the notion that the ‘narrative’ matters when reporting on suicide. Specifically, ‘Werther’ narratives of suicides in celebrities and suicides in non-celebrities where the methods were described were associated with more subsequent suicides while ‘Papageno’ narratives of survival and crisis mastery without depictions of suicidal behaviours were associated with fewer subsequent suicides. These results may inform efforts to prevent imitation suicides.

Suicide reporting may be among the most challenging aspects of journalism ethics given both the media’s duty to inform the public of newsworthy events and known risks of reporting about suicide particularly in identifiable people like celebrities (Cheng et al., 2007; Etzersdorfer et al., 2004; Gould, 2001; Hawton and Williams, 2002; Niederkrotenthaler et al., 2010, 2012, 2020; Pirkis and Blood, 2001; Pirkis et al., 2006b; Sinyor et al., 2018a; Stack, 2003, 2005; Tousignant et al., 2005). Journalists must carefully balance the competing interests of accurately reporting information in a fair and reliable manner, while also mitigating potential negative consequences of their reporting.

Imitative acts of suicide following news reports about suicide deaths, the so-called ‘Werther effect’, may be related to factors such as the extent of media reporting, whether the suicide involves a well-known celebrity (in comparison to non-celebrities), and details of the suicide method (Niederkrotenthaler et al., 2012). A meta-analysis by Stack (2005) established that celebrity suicides were more likely to lead to subsequent ‘copycat’ suicides when compared to non-celebrity suicides. Niederkrotenthaler et al. (2020) demonstrated that there was a 13% increase in suicides in the general public during the weeks following media reporting of a celebrity suicide. One possible mechanism underlying this phenomenon is that individuals strongly relate to the subject of the report through vertical and/or horizontal identification. That is, the risk of ‘copycat’ suicide may increase when a vulnerable person reveres a celebrity (vertical identification) and/or if they see themselves as similar to the celebrity in terms of age, gender and other personal characteristics (horizontal identification) although neither is required for imitation to occur (Hawton and Williams, 2002; Niederkrotenthaler et al., 2009).

Notably, as for maladaptive behaviours, identification can occur in relation to people displaying adaptive behaviours and there may be fewer subsequent suicides when journalists report on stories of resilience and survival, the so-called ‘Papageno effect’ (Niederkrotenthaler et al., 2010; Till et al., 2018).

There is accumulating evidence that the specific suicide content included in media reports and/or the volume of reporting may have a crucial impact on subsequent suicide rates. But individual media reports are not simply a collection of content. They necessarily include a number of specific details that collectively form an overarching story (i.e. the narrative). One of the limitations of prior literature in this area has been a relative dearth of studies focused on narrative. Yet, there is emerging evidence that the impact of overarching story narrative may be of similar or even greater importance than specific elements of content included (Niederkrotenthaler et al., 2010; Sinyor et al., 2021). In their original study coining the term ‘Papageno effect’, Niederkrotenthaler et al. (2010) found that media narratives emphasizing suicidal acts, death and hopelessness were associated with increased subsequent suicides while narratives of mastery over suicidal crises and survival were associated with decreased subsequent suicides. Sinyor et al. (2021) observed results suggesting a similar outcome for social media exposures. Nevertheless, the specific role of narratives remains an underexplored area.

The current study aimed to address this gap by re-examining data from an earlier study by Sinyor et al. (2018b) which found that Werther characteristics of media reports, such as the inclusion of suicide methods and statements that suicide was inevitable, were associated with increased subsequent suicides. The purpose of the current study was to compare reports with Werther and Papageno narrative types in order to identify how this may impact on subsequent suicides. We hypothesize that harmful narratives will be associated with greater subsequent suicides, protective narratives will be associated with fewer subsequent suicides and that narratives not clearly following into either category would be associated with no change in subsequent suicides.

## Method

### Media data

Suicide-related media articles were previously obtained from a media tracking agency (Meltwater Inc.) through an automated keyword search followed by a manual review to confirm relevance (Sinyor et al., 2018b). These articles all had a ‘major focus’ on suicide defined as either having suicide as the main subject of the article or taking up a substantial amount of the text (more than just a few sentences or a small paragraph). Articles were published during 2011–2014 in the 12 most circulated Canadian print and online media publications in the Toronto market and in one US newspaper with broad Canadian readership (see Box 1 and Supplemental Appendix). Articles were coded for a list of putatively harmful and putatively helpful characteristics based on Canadian recommendations for responsible reporting of suicide (Sinyor et al., 2018a). The former included codes for articles that mentioned suicide deaths or attempts in either a celebrity or a non-celebrity, and the presence of suicide methods. The latter included codes for articles that include information about survival and mastery such as examples of positive outcomes of suicidal crisis (e.g. calling a suicide hotline). The coding system demonstrated high inter-rater reliability (Sinyor et al., 2018b). The current analysis combined these variables as a proxy for article narrative. Ideally, articles for such a study would be coded a priori based on standardized definitions for different narratives. As this study relied on data from a previous study that did not have this methodological emphasis, we attempted to reconstruct proxies for narratives based on groupings of content. Specifically, articles were defined as having putatively harmful (‘Werther’) narratives if they (a) described suicide death in a celebrity or (b) described a suicide death in a non-celebrity and included the suicide method. These two definitions were chosen given that they are both likely to include problematic narrative arcs. Articles about celebrity suicide, specifically, were included because these generally follow a narrative of death and hopelessness in someone that many of those exposed will revere and/or identify with (Stack, 2005). Articles about non-celebrity suicide with a description of a method present a story of death that, at least in some way, presents a depiction of the suicide itself rather than simply information that a suicide has occurred.

**Box 1.** Media articles.

• *The Globe & Mail*: theglobeandmail.com (Online and Print) • *The National Post*: nationalpost.com (Online and Print) • *The Toronto Star*: thestar.com(Online and Print) • *The Toronto Sun* • *24 Hours Toronto* • *Maclean’s* • CBC.ca • canada.com • financialpost.com • *The New York Times*

Articles were defined as having putatively protective (‘Papageno’) narratives if they included at least one putatively protective element related to survival and mastery – (a) alternatives to suicides (i.e. seeking treatment), (b) community resource information for suicidal ideation, (c) examples of positive outcomes of suicidal crisis (i.e. calling suicide hotline), (d) messages of hope and (e) information about how to identify and approach a suicidal person – without including any information about suicidal behaviour (attempts or death). Content related to suicidal behaviour was excluded from the Papageno narratives given the aim to capture narratives that did not incorporate the person following through with any form of suicidal act. Note that these two definitions are mutually exclusive. For the purposes of our study, an article could not be categorized as both having Werther and Papageno narratives. Articles with such content as well as any articles that did not meet the criteria specified above were considered as a third control comparator category. Note that 75% of articles from the prior study had content about suicide death and 50% mentioned a suicide method while most putatively protective elements were present in <5% of articles except alternatives to suicide which appeared in 19%. Therefore, as in the study by Niederkrotenthaler et al. (2010), this study represents an examination of a Werther narrative majority of articles compared to a relatively smaller group of outliers that may have Papageno narratives. Articles that did not fall into either narrative definition (e.g. articles about suicide death in non-celebrities with no mention of suicide methods) were excluded from the analyses.

### Suicide data

Data for all deaths ruled as suicide within the city of Toronto, Canada were provided by the Office of the Chief Coroner of Ontario.

### Statistical analysis

The primary outcome variable of interest was the difference in the number of suicide deaths in the week prior to publication (termed week 0; control) and in weeks 1, 2, 3 and 4 after publication (exposure). This duration was chosen as media impacts on suicide rates are frequently observed within the first weeks of an exposure and may often last for a month or more (Fink et al., 2018; Niederkrotenthaler et al., 2020). Note that it was possible for multiple Werther and/or Papageno narrative exposures to occur on the same day or week. Following prior established methods, these were treated as independent events (Sinyor et al., 2018b, 2021). We examined the volume of reports as a continuous variable involving a cumulative count of all articles relating to suicide published between day 0 and day 6 of each time period. We used a latent difference score (LDS) analytical approach (see McArdle, 2001; McArdle and Hamagami, 2001) to examine longitudinal changes in this suicide death variable over time. The advantage of LDS is that non-linear, dynamic change trajectories can be established for any univariate (one variable) longitudinal series, based on the principles of growth curve analysis (Meredith and Tisak, 1990) and cross-lagged regression analysis (Jöreskog and Sörbom, 1979). The model also differentiates the observed score from the associated error in order to evaluate longitudinal changes in the ‘latent’ or ‘error free’ variable over time, providing a more thorough and accurate examination of non-linear longitudinal change in comparison to traditional analytical methods. The first step of LDS modelling always involves considering four possible univariate models (Hamagami and McArdle, 2001; McArdle, 2001; McArdle and Hamagami, 2001). In the *no change* longitudinal model, suicides do not change over time. In the *constant change* longitudinal model, longitudinal change is linear and constant over time – suicides would change based on a constant value across each time period. In the *proportional change* model, longitudinal change is non-linear, and each score is proportional to the previous latent score – e.g. the suicide count at each timepoint would be a proportion of the suicide count from the previous timepoint. In the *dual change* model, latent change is non-linear, involving both additive and proportional changes. The Supplemental appendix provides a more detailed overview of LDS modelling.

Analyses were conducted using the AMOS 25.0 programme (Arbuckle, 2011) and parameters were estimated using the maximum-likelihood method. Indices of absolute and relative model fit were considered when evaluating models. The root mean square error of approximation (RMSEA) (Steiger, 1998) is an indicator of model discrepancy in comparison to the degree of freedom. RMSEA values of 0.5 or less indicate a ‘close fit’ (Browne and Cudeck, 1993). The chi-square (χ^2^) index of absolute model fit was assessed. According to Byrne (2004), chi-square to degrees of freedom ratio values (χ^2^*/df*) below two are considered acceptable although Schermelleh-Engle et al. (2003) propose that a ratio between 2 and 3 is indicative of a ‘good’ or ‘acceptable’ data-model fit, respectively. The Akaike information criterion (AIC) (Akaike, 1973) is an indicator of relative model fit that considers model complexity relative to the number of parameters; the model with smaller AIC is preferred. The comparative fit index (CFI) was assessed; CFI values greater than 0.90 indicate a ‘good fit’ when comparing models (CFI) (Bentler, 1990).

## Results

### LDS analyses: Univariate, multigroup (comparing Werther vs Papageno narratives)

Correlations for the full dataset (6367 articles and suicides weeks 0–4) without differentiating based on narrative themes are presented in Supplementary Tables 1–3.

**Figure 1.**
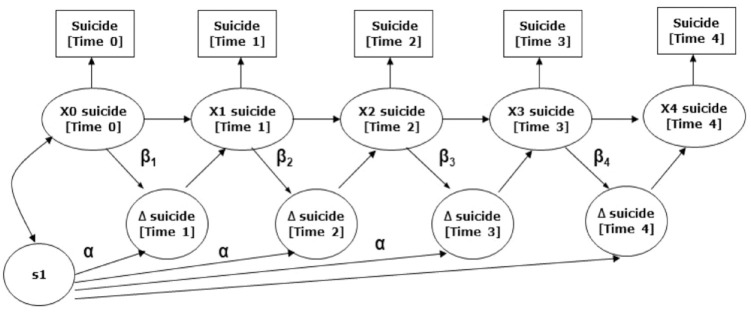
Path diagram of the suicide univariate LDS model, illustrating the longitudinal change in death count over time. This diagram is used to illustrate longitudinal trajectories of suicide over time. Squares represent observed variables. Circles represent latent variables. Single-headed arrows represent regression coefficients. Suicidality[*t*] represents the cumulative death count for articles examined at time *t*. suicidality[*t*] represents the associated latent scores at time *t. e*(*t*) represents the error term at time *t*. (α × *s_n_*) represents a fixed slope score. β(*t*) indicates the time-varying proportional effect. Time 1 = Week 0 (prior to publication); Time 2 = Week 1 (after publication); Time 3 = Week 2 (after publication); Time 4 = Week 3 (after publication); Time 5 = Week 4 (after publication). Interested readers who would like an overview of LDS modelling can refer to Hawley et al. (2006) for further information.

LDS univariate analyses considered four models, consisting of the *no-change* model, the additive *constant change* model, the *proportional change* model and the combined *dual change* model. Both time-varying and time-invariant proportional effects (β) were considered. Examination of parameter significance and goodness-of-fit indices indicated that changes in suicides over time was best represented as a dual change model ((χ^2^ [2], *N* = 6367) = 3.66; χ^2^/*df* = 1.83; AIC = 60.06, CFI = 0.93, RMSEA = 0.05). All parameter estimates were statistically significant (*p* < 0.05). See Figure 1 for the path diagram for this suicide model.

The α and β coefficients resulting equation characterizes the change in suicide using two components: additive change (i.e. α_suicide_ × *s*_suicide,*n*_) and proportional change, i.e. β_suicide_ × suicide (*t* − 1). According to the modelling results, an equation can be generated that describes the longitudinal model based on each of the variables

$$\begin{array}{l} \Delta \mathrm{Suicide}{\left(t\right)}_{n}=\alpha_{\mathrm{suicide}}\times E\left[s_{\mathrm{suicide},n}\right]+\beta_{s}\\ \times E\left[\mathrm{Suicide}\;{\left(t-1\right)}_{n}\right] \end{array}$$

**Figure 2.**
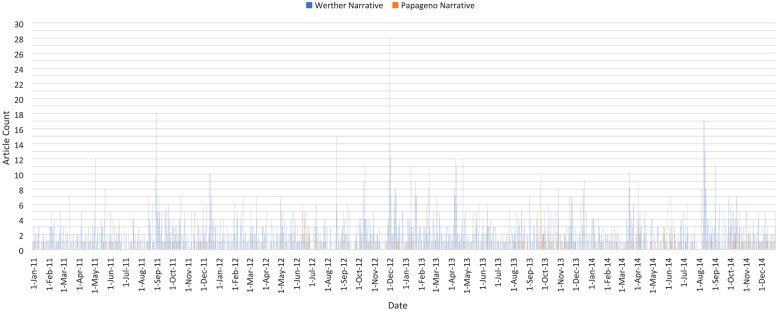
Occurrence of articles with Werther and Papageno narratives in the Toronto media market (2011–2014).

Next, a multigroup model was considered, comparing articles classified as having Werther and Papageno narratives. Among the Werther narrative articles, there were *N* = 662 articles that mentioned a celebrity death, and *N* = 2220 that mentioned the death of any non-celebrity, with any suicide method (i.e. 2882 total Werther narrative articles). For the Papageno narrative articles, there were *N* = 355 articles that did not mention any suicide attempt or death and involved a protective element. Note that yearly counts of Werther narrative articles were relatively stable from 2011 (655) to 2014 (713) while the number of Papageno narrative articles increased substantially from 2011 (54) to 2014 (130) (Figure 2).

**Figure 3.**
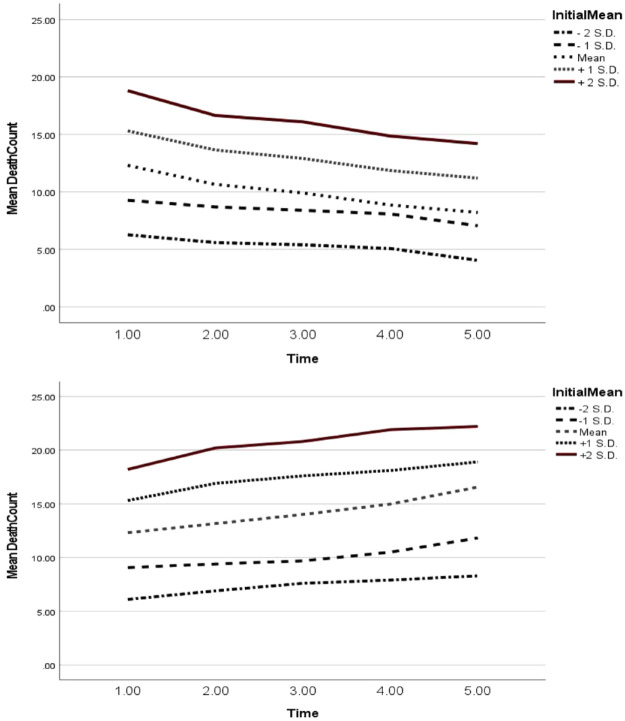
Estimated change trajectories for the Papageno (top) and Werther (bottom) groups, based on the LDS equation,using (a) initial Time 1 mean, (b) one standard deviation above the initial mean, (c) two standard deviations above the initialmean, (d) one standard deviation below the initial mean and (e) two standard deviations below the initial mean. The X-axis represents each timepoint: Time 1 = Week 0 (prior to publication); Time 2 = Week 1 (after publication); Time Week 2 (after publication); Time 4 = Week 3 (after publication); Time 5 = Week 4 (after publication).

The multigroup analysis demonstrated significant goodness of fit and parameter estimates and demonstrated that the Werther narrative group differed from the putatively protective narrative group, with each group demonstrating different trajectories of change in reported suicides over time: (χ^2^[6], *N* = 6367) = 13.16; χ^2^/df = 2.19; AIC = 97.16, CFI = 0.96, RMSEA = 0.03. Overall, the Papageno narrative group trajectories declined significantly over time in comparison to the Werther narrative group trajectories, which either increased or plateaued. In Figure 3, trajectories are plotted based on various levels of initial suicide. For example, in the top graph of Figure 3, considering the Papageno narrative condition, in each trajectory, the cumulative death count decreases over time. In the bottom graph of Figure 3, considering the Werther narrative condition, the cumulative death count increases over time and the magnitude of this impact increases as the initial death count increases. For example, if there is a low initial suicide count (2 standard deviations below the sample mean), there is a small increase in subsequent suicides over time as compared to a high initial suicide count (2 standard deviations above the sample mean), and there is a larger increase in subsequent suicide counts over time.

We examined the remaining articles (*N* = 3130) that were not categorized as containing either the Papageno or Werther narratives. The resulting model demonstrated acceptable indices of absolute and relative model fit: (χ^2^[10], *N* = 3130) = 19.87; χ^2^/df = 1.99; AIC = 102.38, CFI = 0.95, RMSEA = 0.04. Considering the resulting trajectory, these articles demonstrated minimal change over time.

## Discussion

The results of our longitudinal modelling demonstrate that the trajectory of suicides following protective ‘Papageno’ narrative reports declined significantly over time, while those following putatively harmful ‘Werther’ narrative reports increased with no substantial change following reports with neither of these narratives. These findings coincide closely with our understanding of social learning and imitative behaviour as well as our a priori hypotheses. The results of recent research related to suicide and the media, including the present study, suggest that further attention ought to be given to the content and messages embedded within the overarching narratives of such stories. Although we acknowledge that stories of celebrity suicides will always be newsworthy, our LDS modelling results demonstrate that media reports can have differing impacts on suicide rates across a population depending on the narrative. These narratives are also relevant when examining other forms of media. For example, the protective ‘Papageno’ effect was illustrated in a recent study examining the impact of the song ‘1-800-273-8255’ by hip-hop artist Logic; this song provided a phone number for an emergency service called ‘Lifeline’, and this was associated with a large increase in calls to the service (Niederkrotenthaler et al., 2021). A reduction in suicides was observed during the time periods with the most social media discourse about the song. The harmful ‘Werther’ effect was examined as related to the Netflix show ‘*13 Reasons Why’* (Niederkrotenthaler et al., 2019). The results indicated associations between the show and subsequent suicide counts among its young target audience.

These findings are highly relevant for the development of future responsible media reporting guidelines as existing guidelines have thus far focused mainly on recommendations about specific story content rather than overarching narrative (Pirkis et al., 2006a; World Health Organization and International Association for Suicide Prevention, 2017). This is important and must continue given that careful attention to the specific content included may serve to mitigate some harms and potentially confer some protection in the context of suicide stories in the media. However, those developing guidelines ought to both consider a greater emphasis on the contribution of overarching narrative and a shift from a main emphasis on mitigating negative effects towards both content *and narratives* that may facilitate positive ones. Specifically, this study suggests that a greater emphasis on incorporating Papageno narratives while avoiding Werther narratives is likely to prevent some subsequent suicides. Media professionals should be encouraged to present narratives of hope and survival, telling stories of people who do not engage in suicidal behaviour/do not die by suicide and instead engage in adaptive coping mechanisms in order to overcome challenging situations. Unfortunately, reporting emphasizing suicide death in identifiable people and/or with descriptions of suicide methods can have negative consequences, leading to imitative behaviour among vulnerable individuals. While stories with narratives of death will continue to be published, it is possible that, as with specific content, subtle differences in the narrative frame of such stories (e.g. a missed opportunity for help-seeking rather than an inevitable consequence of life stress) could help to mitigate risk across the population. Ideally, stories of hope and survival would be the norm rather than the exception. Journalists are encouraged not only to report on suicides but also to seek out and report on stories of mastery and survival, which may have a meaningful, positive influence on suicides across a population. Given this goal, the fact that the number and proportion of Papageno narrative articles increased over time is an encouraging finding.

Our results are in line with recommendations in guidelines for media reporting such as the World Health Organization and International Association for Suicide Prevention (WHO/IASP, 2017) guidelines. These guidelines suggest that responsible reporting should involve ‘protective’ elements including how individuals can overcome suicidal ideation, which may help to promote effective coping and resiliency over the longer term. Our findings suggest that future iterations of such guidelines place an even greater emphasis on the importance of the overarching narrative arc of media reports on suicides across the population. This may be particularly relevant to consider this early in the process, when the narrative is being developed (e.g. for those in the entertainment industry). For them to have an impact, it would also be important to ensure robust knowledge translation efforts so that guidelines and their evidentiary basis are disseminated widely to the journalism community.

A somewhat unexpected finding of our study was that the strength of the potential Werther effect (and to a lesser extent the Papageno effect) seemed to increase by the fourth week after media item publication. One possible explanation for this is that the results reflect a type 1 error (i.e. that these changes were unrelated to media articles). While we know that the effects of media reports on population level suicide rates are often highest in the 1–2 months after publication (Niederkrotenthaler et al., 2020), to our knowledge, there are no studies that have systematically examined if such effects always peak in the first weeks after a potential exposure. While we might expect media articles to have relatively immediate, transient effects, on people exposed to them during an acute suicidal crisis, we also know that effects can occur over longer time scales. For example, the number of excess suicides in the United States following Robin Williams’ suicide, which occurred on 11 August 2014, was numerically higher in September 2014 than in August 2014 (Fink et al., 2018) and this is consistent with our observation of larger increases 4 weeks later. Further research characterizing, at a more granular level, the duration and pattern of Werther and Papageno effects would be helpful.

This study has strengths as well as limitations. Strengths include a comprehensive review of all available print media items in selected newspapers including suicide content over a lengthy period of time (4 years), with good inter-rater reliability, which speaks to the potential validity, reliability and generalizability of these results. The novel use of the LDS statistical approach is also a strength, as this statistical approach examines non-linear, dynamic change over time, based on latent change (this model separates error from the latent ‘error free’ version of each variable).

In terms of limitations, this study did not examine narratives in a priori fashion but rather constructed proxies for harmful and protective narrative types using collections of individual article characteristics collected for a previous study. There was insufficient data to expand those constructed narratives to include more nuanced article types such as those presenting putatively harmful elements (e.g. the description of a suicide method) in the context of narratives of mastery and survival or, conversely, putatively protective elements (e.g. crisis resource information) in the context of a narratives of death and despair. Furthermore, because Werther stories are specifically known to exert powerful effects (Niederkrotenthaler et al., 2020) and because both Papageno elements and narratives were rare in our data, we set up our analysis as a dichotomy in which the presence of a Werther narrative trumped the presence of a Papageno one. This was done to assist in identifying the potential impact of ‘pure’ Papageno narratives. However, as efforts to educate the media continue, we expect more news stories to include both types of narratives and investigating the potential impact of such stories is also an important target of future work. This would be a useful question for future research as would the relative contribution of overarching narrative and specific story elements. In general, we acknowledge that this study examined relatively blunt distinctions for what constitute Werther and Papageno narratives and this was due to limitation in the available data. Subsequent research should include of more detailed and varied narrative-related information to help advance our understanding of this emerging research area. Note that we also followed prior methods treating each media item as independent events (Sinyor et al., 2018b, 2021). This strategy has advantages as, if our hypothesis is correct, we would expect to observe more suicides after weeks where there were many Werther narratives articles and fewer suicides after weeks where there were more Papageno narrative articles. However, this methodology is unable to identify the impact of narratives of different salience (i.e. depending on the details, some may have a greater emotional impact than others) or whether having a certain volume of concurrent Werther or Papageno narratives might counteract or interfere with each other’s impact. Furthermore, specific characteristics of individuals who died by suicide that might have affected personal vulnerability were not examined. This methodology did not allow us to demonstrate that those who died by suicide were exposed to specific media reports. This is an inherent limitation of all studies of this phenomenon. These studies permit an analysis of the most relevant outcomes to suicide prevention, suicide and self-harm, but they do not allow for causality to be inferred. Furthermore, this study only examined print and online reports, although it is likely that these have substantial overlap with television news stories and possibly with some social media content. The relative impact of these related media on the phenomena observed could be the subject of future research. Finally, it is important to acknowledge that media reporting is only one of a myriad of factors that influence suicide at a population level and that our analysis did not account for other potential confounders. Although we are not aware of any events that might have impacted the specific time periods studied across media items, we cannot rule out that possibility and therefore our results do not establish causality, must be interpreted with caution and are in need of replication.

In conclusion, these results are consistent with previous analyses examining the impact of media reporting of suicide. Since the narrative matters, there is a need for journalists to consider recommendations for responsible reporting such as the WHO/IASP guidelines when reporting on suicide, in order to help save lives. Considering future directions, it would be informative to replicate these findings with an a priori methodology that captures narrative in a more direct and nuanced way. Future research may also examine whether recommendations about media narratives are effective and to understand any barriers to their implementation. Furthermore, it may be helpful to understand how the Werther and Papageno effects differentially impact on individuals based on the type of media consumed, comparing the relative impact of traditional media (e.g. newspapers, magazines) to other formats (e.g. television, various forms of social media, podcasts, vlogs). Regardless, these results underscore the potential underappreciated importance of narrative in social learning and suicide prevention and deserve further attention by researchers and media content creators.

## Supplemental Material

sj-pdf-1-anp-10.1177_00048674221117072 – Supplemental material for Is the narrative the message? The relationship between suicide-related narratives in media reports and subsequent suicides Click here for additional data file. Supplemental material, sj-pdf-1-anp-10.1177_00048674221117072 for Is the narrative the message? The relationship between suicide-related narratives in media reports and subsequent suicides by Lance L Hawley, Thomas Niederkrotenthaler, Rabia Zaheer, Ayal Schaffer, Donald A Redelmeier, Anthony J Levitt, Jitender Sareen, Jane Pirkis and Mark Sinyor in Australian & New Zealand Journal of Psychiatry
