# Supplementary material for: Is the narrative the message? The relationship between suicide-related narratives in media reports and subsequent suicides
Source: Aust N Z J Psychiatry. 2022 Aug 23;57(5):758–66. doi: 10.1177/00048674221117072 (PMC10126449; doi:10.1177/00048674221117072)
Supplement: sj-pdf-1-anp-10.1177_00048674221117072 – Supplemental material for Is the narrative the message? The relationship between suicide-related narratives in media reports and subsequent suicides [file sj-pdf-1-anp-10.1177_00048674221117072.pdf]

Appendix

Overview - Latent Difference Score Analysis

Table 1 presents means, standard deviations, and correlations among study variables at week 0 and weeks 1, 2, 3, and 4, after the publication of the article. Each column provides information regarding correlations among the study variables. At the bottom of each column, the mean number of suicide deaths and standard deviation is provided for week 0 (one week prior to publication), week 1 (one week after publication), week 2 (two weeks after publication), week 3 (three weeks after publication) and week 4 (four weeks after publication). These means represent the entire sample data, without differentiating based on the narrative themes. The mean number of suicide deaths variable demonstrated relatively non-linear change over time. There were positive correlations between measures from consecutive assessments for each measure. Data from 6367 articles were examined.

In the LDS modeling framework, an observed score (e.g., number of suicides, $Suicide(t)_n$) from an article (*n*) at time *t* can be decomposed into a latent or "true" score ($suicide(t)_n$) considered to be measurement error-free as well as the associated independent measurement error ($e(t)_n$). This is shown as: $Suicide(t)_n = suicide(t)_n + e(t)_n$. Measurement errors are assumed to have a mean of zero ($\mu_e = 0$), to have nonzero variance (σ^2_e), to be uncorrelated with other error terms in the model, and to have the same variance at each time point. The difference between consecutive latent scores is expressed as a "latent difference score": ($\Delta Suicide(t)_n = Suicide(t)_n - Suicide(t - 1)$). In this equation, the latent suicide score from an article *n* at time t is the sum of the latent score at the previous time point (t-1), and

the latent difference score, $\Delta Suicide(t)_n$. If observations occur at fixed intervals, the time between pairs of latent scores is constant (i.e., $\Delta t = 1$) and the latent difference score can be interpreted as the rate of change of the true score ($\Delta Suicide(t)_n/\Delta t = \Delta Suicide(t)_n$.)

Univariate LDS Models

Using the latent rate of change ($\Delta Suicide(t)_n/\Delta t$) as the outcome variable, there are several ways to model univariate longitudinal change(Hamagami and McArdle, 2001; McArdle and Nesselroade, 2003). In the "dual change" model, suicide change over time can be expressed as: $\Delta Suicide(t)_n = \alpha_{Suicide} \times s_{Suicide,n} + \beta_{Suicide} \times Suicide\,(t - 1)_n$ .The additive change component ($\alpha_{Suicide} \times s_{Suicide,n}$) involves change which is constantly related to a score $s_{Suicide,n}$. This is a latent variable, involving values that vary across subjects but are constant over time. The coefficient $\alpha_{Suicide}$ can be considered as a factor loading, and is usually fixed to 1 for identification purposes. The $s_{Suicide,n}$ term is the intercept term which can change from subject to subject. The proportional change component ($\beta_{Suicide}$ x Suicide $(t - 1)_n$) involves change that is proportional to the previous latent score. $\beta_{Suicide}$ indicates the proportional effect of a previous latent variable on the subsequent rate of change, and can be either time-invariant, or time-varying (i.e., $\beta_{Suicide}(t)$).

Simplifying the dual change model leads to three models of univariate change. In the *constant change score* model, the $\beta_{Suicide}$ coefficient is set to zero, and latent change is constant within a subject over time.

The resulting equation would be: $\Delta Suicide(t)_n = \alpha \times s_{Suicide,n}$, $\beta_{Suicide} = 0$

In the *proportional change score* model, the $\alpha_{Suicide}$ coefficient is set to zero, and latent change is proportional to the latent score from the previous time point.

The resulting equation would be: $\Delta Suicide(t)_n = \beta_{Suicide} \times Suicide\,(t - 1)_n$, $\alpha_{Suicide} = 0$

In the *no change score* model, the latent scores do not change over time. However, the observed scores may vary over time due to the random error term, $e(t)_n$. The resulting equation would be: $\Delta Suicide(t)_n = 0$, $\alpha_{Suicide} = \beta_{Suicide} = 0$

Figures 1a, 1b, 1c and 1d represent the four univariate LDS models as path diagrams used in structural equation modelling. In these path diagrams, a latent variable (represented as a circle) is comprised of an observed variable (represented as a square), with associated measurement error (represented as a circle). A directional arrow symbolizes the influence of one latent variable on another, while a bi-directional arrow represents correlations.

The resulting equation characterizes the change in Suicide using two components: additive change (i.e., $\alpha_{suicide}$ x $s_{suicide,n}$), and proportional change (i.e., $\beta_{suicide}$ x suicide (t - 1). Based on the modelling results, an equation can be generated that describes the longitudinal model based on each of the variables: $\Delta Suicide(t)_n = \alpha_{suicide} \times E[s_{suicide,n}] + \beta_s \times E[Suicide\ (t-1)_n]$

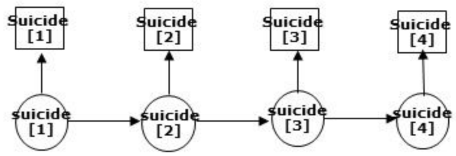

a) No Change Model

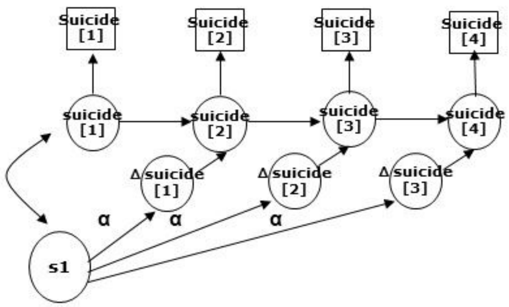

b) Constant Change Model

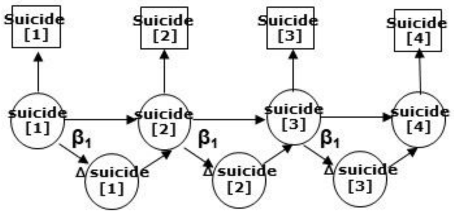

c) Proportional Change Model

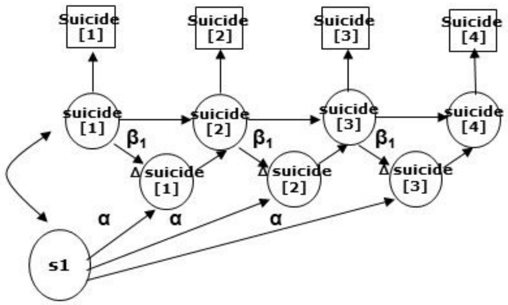

d) Dual Change Model

Table 1

Correlations, Means and Standard Deviations for Study Measures (Full Sample)

Variable	1. Suicide $_{t0}$	2. Suicide $_{t1}$	3. Suicide $_{t2}$	4. Suicide $_{t3}$	5. Suicide $_{t4}$
1. Suicide $_{t0}$	1.00	---	---	---	
2. Suicide $_{t1}$	-0.10**	1.00	---	---	
3. Suicide $_{t2}$	-0.67**	-0.14**	1.00	---	
4. Suicide $_{t3}$	0.05**	-0.05**	-0.16**	1.00	
5. Suicide $_{t4}$	0.04**	0.04**	0.01	-0.12**	1.00
M	9.08	9.09	9.07	9.01	9.15
SD	3.04	2.91	2.89	2.98	2.99

Notes: N = 6367. Suicide = Number of suicide deaths; t0 = Week 0 (prior to publication); t1 = Week 1 (after publication); t2 = Week 2 (after publication); t3 = Week 3 (after publication); t4 = Week 4 (after publication); *M* = Mean, *SD* = Standard deviation.
* $p < .05$. ** $p < .01$.

Table 2: *Univariate Suicidality Analyses*

Parameters and Fit Indices	No change	Constant Change	Proportional Change	Dual Change
Additive coefficient				
$E(s_n)$	0 (=)	-14.11[a]	0 (=)	-17.26[a]
σ^2 (s_n)	0 (=)	5.19	0 (=)	5.93
Proportional coefficients				
β_a	0 (=)	0 (=)	0.11	1.86[c]
β_b	0 (=)	0 (=)	0.15	1.88[c]
β_c	0 (=)	0 (=)	0.28	1.91[c]
Goodness-of-fit indices				
Parameters	6	9	9	12
Degrees of Freedom	8	5	5	2
RMSEA (p close fit)	0.11(.01)	0.18(.01)	0.09(0.02)	0.05(.39)
CFI	0.46	0.74	0.47	0.93
AIC	264.75	81.26	62.57	60.06
χ^2	52.76	63.26	34.57	3.66
χ^2/df	6.60	12.65	6.91	1.83

Note. 0 (=) indicates parameter is not estimated. "*p* close fit" = *p* value for testing the null hypothesis that the population RMSEA is not greater than .05; CFI = comparative fit index; AIC = Akaike information criterion; E(*sn*) = additive change coefficient; β = proportional change coefficient.
[a] $p < .05$. [b] $p < .01$. [c] $p < .001$.

Table 3: *Multigroup Suicide Analysis Comparing Articles with Harmful vs. Protective Narratives*

Parameters and Fit Indces	Harmful (Werther) Narrative Group	Protective (Papageno) Narrative Group
Additive coefficient		
$E(s_n)$	-1.12[a]	3.21[a]
σ^2 (s_n)	0.26	1.17
Proportional coefficients		
β_a	0.16[a]	-0.42[a]
β_b	0.15[a]	-0.39[a]
β_c	0.16[a]	-0.44[a]
βd	0.19[a]	-0.42[a]
Goodness-of-fit indices		
Parameters	34	
Degrees of Freedom	6	
RMSEA (p close fit)	.03 (.98)	
CFI	.96	
AIC	97.16	
χ^2	13.16	
χ^2/df	2.19	

Notes.

1. *Note.* Suicidality = Number of suicide deaths reported. 0 (=) indicates parameter is not estimated. "*p* close fit" = *p* value for testing the null hypothesis that the population RMSEA is not greater than .05; CFI = comparative fit index; AIC = Akaike information criterion. E(*sn*) = additive change coefficient; β = proportional change coefficient. The α and β coefficients resulting equation characterizes the change in Suicide using two components: additive change (i.e., $\alpha_{suicide}$ x $s_{suicide,n}$), and proportional change (i.e., $\beta_{suicide}$ x suicide (t - 1). Based on the modelling results, an equation can be generated that describes the longitudinal model based on each of the variables: $\Delta Suicide(t)_n = \alpha_{suicide} \times E[s_{suicide,n}] + \beta_s \times E[Suicide\ (t-1)_n]$
2. ^{a} $p < .05$. ^{b} $p < .01$. ^{c} $p < .001$.
